# Colds

**Published:** 1880-12

**Authors:** 


### COLDS.

A cold in the head can usually be cured in a few hours, if, as soon as it is discovered, the person will sniff the fumes of ammonia or spirits of camphor every few minutes as strong as they can be borne. When a severe cold attacks the throat and lungs, there is no safety in neglecting it for an hour, for there is more or less inflammation of the lungs, which interferes with their action, rendering them liable at any moment to take on serious and, possibly, uncontrollable disease. Go home and remain there. In the evening take a warm foot bath, and at bed time take 3 or 4 liver pills. These will stimulate the liver to healthy action, and promptly relieve the lung trouble ; but it renders the system sensitive to renewed attacks, and, therefore, the greatest care should be observed for several days not to take fresh colds. Ordinarily, no other treatment is necessary ; but, should the cough continue, have your druggist make the following mixture, and take 1 or 2 teaspoonsful every hour till cured : Glycerine, 4 ounces ; whiskey, 4 ounces ; morphine, 1 grain.

Sore throat can be promptly relieved by applying a mustard plaster, or "mustard leaves," on the front of the neck, over the sore spot. In addition the throat and mouth may be frequently gargled with the following mixture :

A teaspoonful of salt, a pinch of red pepper, and a tablespoonful of vinegar. If found too strong, add a little water. A portion of the gargle may be swallowed, or sipped, little at a time.

Sweating has been quite generally recommended, in the cure of colds. I think, however, that the risk of taking cold afterward will more than counterbalance the good that may be expected, and, except in cases of unusual severity, I would not recommend it.

The "Turkish Bath" has been highly recommended as a cure for colds. With proper care afterward, there is no better or more effectual plan of cutting short a severe cold. It is not, however, necessary to go to a regular establishment to take such a bath. Any small comfortable room, where a good fire can be quickly made, will answer the purpose. In the early evening make a good fire in the stove and close the doors and windows, leaving only small openings for ventilation. Then let the patient put on his night clothes and lie down on a bed or sofa, or sit in an easy chair for an hour or more, with the temperature of the room from 90° to 100° F. Afterward the room should be gradually cooled to about 70°, and the patient should go to bed and remain there till morning.

